# Supplementary material for: Efficiently Anti-Obesity Effects of Unsaturated Alginate Oligosaccharides (UAOS) in High-Fat Diet (HFD)-Fed Mice
Source: Mar Drugs. 2019 Sep 17;17(9):540. doi: 10.3390/md17090540 (PMC6780860; doi:10.3390/md17090540)
Supplement: Supplementary file 1 [file marinedrugs-17-00540-s001.pdf]

Table S1 ingredient composition of the experimental diets.

	STD	HFD
Carbohydrate (% of energy)	64.00	42.00
Protein (% of energy)	20.00	17.00
Fat (% of energy)	7.00	41.00
Energy (kcal/kg)	4000.00	4701.00
**Ingredient, g/kg**		
Casein (form milk)	200.00	195.00
Corn starch	397.49	150.00
Sucrose	100.00	341.46
Dextrose	132.00	0.00
Cellulose	50.00	50.00
Soybean oil	70.00	0.00
Milk fat	0.00	210.00
Mineral mixture	35.00	35.00
Vitamin mixture	10.00	10.00
TBHQ	0.01	0.04
DL -Methionine	0.00	3.00
L -Cystine	3.00	0.00
Calcium carbonate	0.00	4.00
Cholesterol	0.00	1.50
Choline bitartrate	2.50	0.00
Chokeberry powder	0.00	0.00
Total	100.00	100.00

Table S2. The primers sequences used for PCR reaction.

Gene name	Primers sequences
SREBP-1c	Forward: 5'-ATCCTGGCCACAGTACCACT-3'<br>Reverse: 5'-GGAACGGTAGCGCTTCTCA-3'
FAS	Forward: 5'-TCGACTTCAAAGGACCCAGC-3'<br>Reverse: 5'-ACTGCACAGAGGTGTTAGGC-3'
ACC	Forward: 5'-AGTGATGGTGGCCTGCTCTTG-3'<br>Reverse: 5'-AGCAGACGGTGAGCGCATTA-3'
HMGCR	Forward: 5'-CCTCCATTGAGATCCGGAGGA-3'<br>Reverse: 5'-ACAAAGAGGCCATGCATACGG-3'
PPARγ	Forward: 5'-TGGGGATGTCTCACAATGCC-3'<br>Reverse: 5'-AGACTCTGGGTTCAGCTGGT-3'
C/EBPα	Forward: 5'-AGGCCAAGAAGTCGGTGGATA-3'<br>Reverse: 5'-TCACTGGTCAACTCCAACACC-3'
Adiponectin	Forward: 5'-TGGAATGACAGGAGCGGAAG-3'<br>Reverse: 5'-GCGAATGGGAACATTGGGGA-3'
PLIN	Forward: 5'-GTCAATGAACAAGGGCCCAAC-3'<br>Reverse: 5'- CACAGGCAGCTGCAGAACTCTC-3'
HSL	Forward: 5'-TCCTGGAACTAAGTGGACGCAAG-3'<br>Reverse: 5'- CAGACACACTCCTGCGC ATAGAC-3'
FABP4	Forward: 5'-TGGGAACCTGGAAGCTTGTCTC-3'<br>Reverse: 5'- GAATTCCAC GCCCAGTTTGA -3'

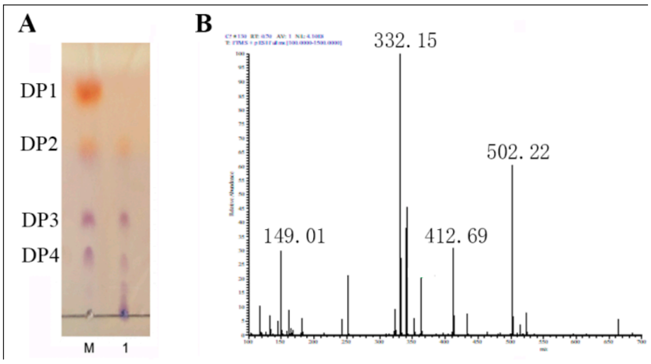

Figure S1 TLC analysis (A) and positive-ion ESI-MS analysis (B) of prepared COS.

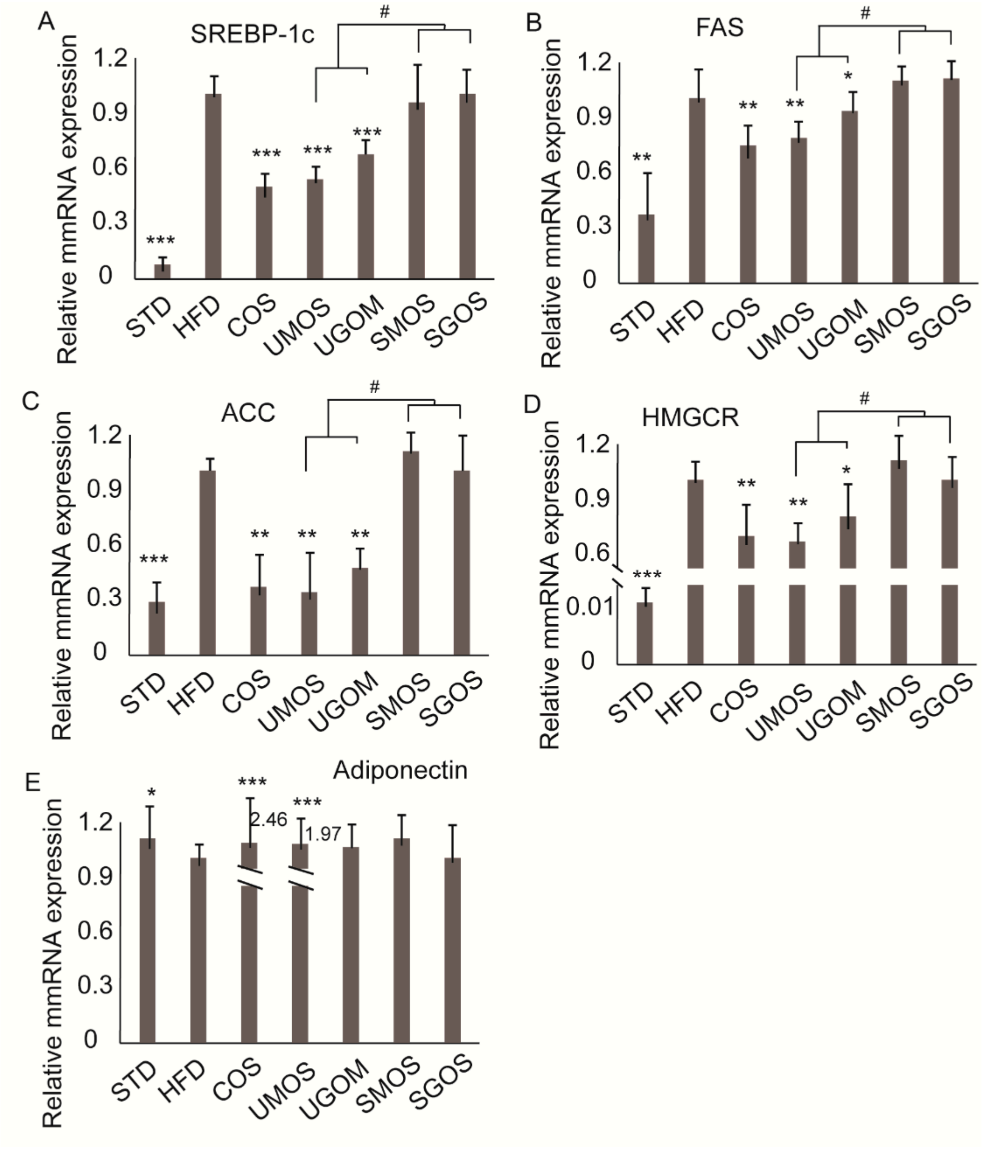

Figure S2. Effect of UAOS on the expression of lipid-related genes in liver. The liver mRNA expression levels of SERBP1-1c (A), FAS (B), ACC (C), HMGCR (D) and Adiponectin (E) were detected by Q-PCR. The data are represented as means ± standard deviation (SD, n =12). Compare with HFD group, * $p < 0.05$, ** $p < 0.01$, *** $p < 0.001$; Compare groups as indicated, $^{\#}p < 0.05$, $^{\#\#}p < 0.01$.

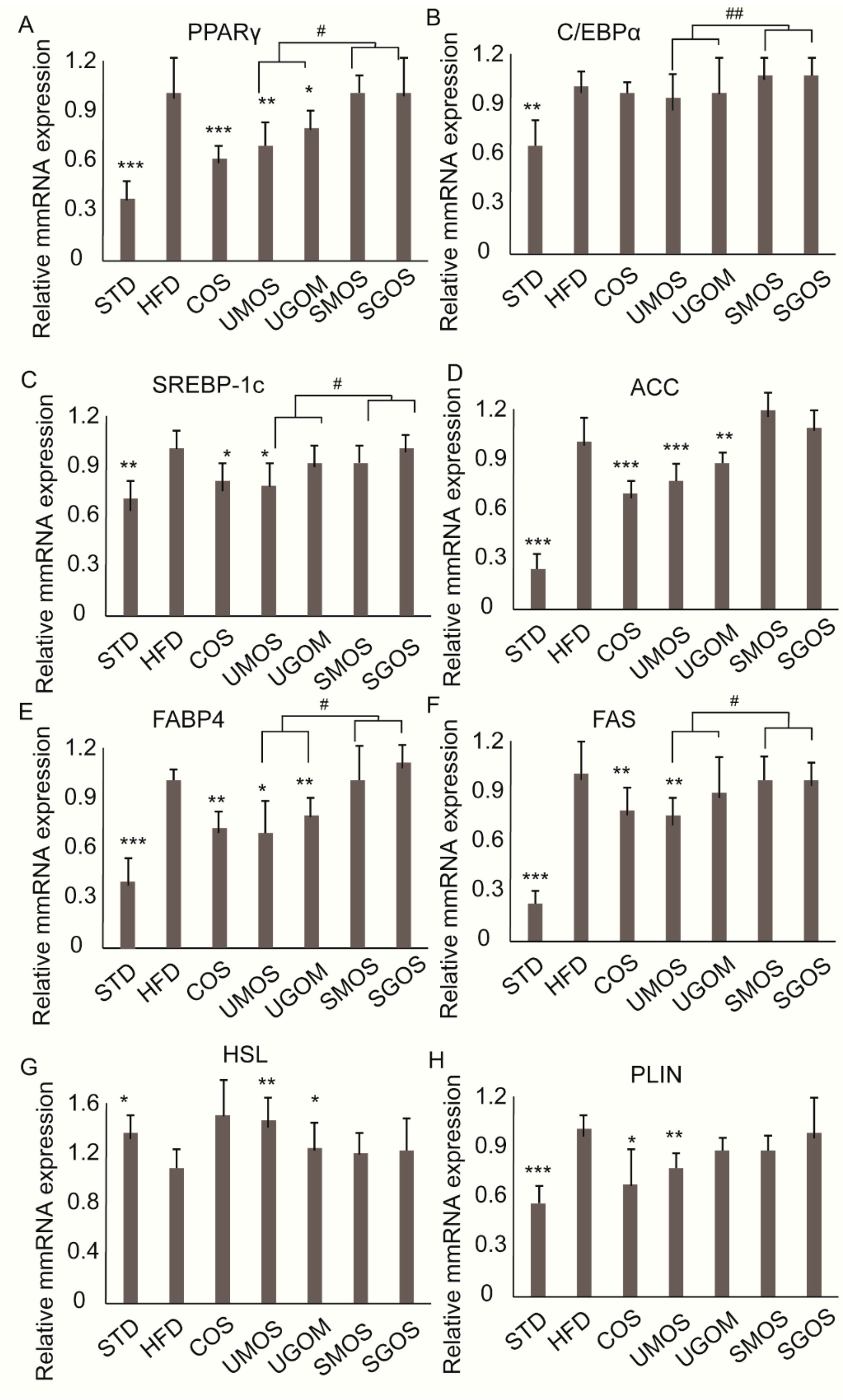

Figure S3. Effect of UAOS on the expression of lipid-related genes in epididymal WAT. The epididymal WAT mRNA expression levels of PPARγ(A), C/EBPα (B), SERBP1-1c (C), ACC (D), FABP4 (E), FAS (F), HSL (G) and PLIN (H) were detected by Q-PCR. The data are represented as means ± standard deviation (SD, n =12). Compare with HFD group, * p < 0.05, ** p < 0.01, *** p < 0.001; Compare groups as indicated, # p < 0.05, ## p < 0.01.
